# Longevity of Anterior Composite Restorations for Localized Tooth Wear: A Scoping Review

**DOI:** 10.3390/dj11110255

**Published:** 2023-10-31

**Authors:** Sindhu Rajarajan, Neil Nathwani, Touraj Nejatian, Peter Fine, Albert Leung

**Affiliations:** Department of Continuing Professional Development, UCL Eastman Dental Institute, Rockefeller Building, 21 University Street, London WC1E 6ED, UK; n.nathwani@ucl.ac.uk (N.N.); t.nejatian@ucl.ac.uk (T.N.); p.fine@ucl.ac.uk (P.F.); albert.leung@ucl.ac.uk (A.L.)

**Keywords:** tooth surface loss, anterior composite restorations, direct and indirect composites

## Abstract

(1) Objective: This scoping review evaluates composite restorations as a treatment modality for anterior tooth surface loss and investigates the longevity of the direct and indirect composites used herein. (2) Method: The search encompassed Medline, Embase, Web of Science, the Cochrane Library, and hand search utilizing the PICO framework. (3) Results: Eight studies were included in this review, comprising one randomized controlled trial, one retrospective, and six prospective studies. Some studies reported favorable outcomes for composite restorations in anterior teeth. Although not statistically significant, evidence supported the anterior composite as a viable short- to medium-term solution for managing tooth wear. Direct resin composites were deemed clinically and cost-effective when managing localized anterior tooth wear. However, limitations and inconsistencies in this scoping review limited definitive clinical recommendations. (4) Conclusions: Further research, including well-managed randomized controlled trials using standardized protocols and longer follow-up periods, is essential to reconfirm the long-term efficacies of anterior composite restorations when managing tooth wear. A robust research design and exacting protocols could facilitate more meaningful clinical conclusions.

## 1. Introduction

There has been a notable trend in the adult population retaining their natural teeth for longer [1], whilst the younger population tends to consume a higher quantity of erosive beverages [2]. These trends are attributed to an increased occurrence of tooth surface loss, encompassing the gradual erosion of dental hard tissues unrelated to caries, trauma, or developmental anomalies [3]. As a natural physiological occurrence, it progresses irreversibly with age, which results in an estimated vertical enamel loss of between 20 and 38 μm per annum [4].

Tooth wear becomes pathological if it surpasses that anticipated for the patient’s age, imperiling its prognosis [2].

There is growing evidence that pathological tooth wear is becoming more prevalent, particularly among young individuals. There is widespread agreement among dental practitioners that severe tooth wear has multifactorial etiologies. Extrinsic and intrinsic erosive acids, abrasion, and attrition can all simultaneously contribute to etiological factors in tooth wear [3,5]. All four modes of tooth surface loss (TSL), namely attrition, abrasion, erosion, and abfraction, are well-defined in the literature [5]. Understanding the underlying causes of tooth wear is critical to reaching the appropriate diagnosis; this facilitates optimal management, including prevention, the education of patients, the use of treatment planning, and the selection of the most suitable appropriate restorative materials where indicated. While anterior TSL often prompts patient seeking advice, posterior TSL frequently goes unnoticed by patients, emphasizing the dentist’s role in early detection [2].

Preventing TSL is most desirable as restorative procedures can be complex, sub-optimal, ongoing, and costly [6]. Identifying and eliminating these etiological factors by instigating an active preventive regime is clearly most desirable.

To monitor the severity and progression of tooth wear, various indices have been proposed, including the widely-used tooth wear index (TWI) by Smith and Knight [7]. However, TWI does not correlate with the cause and consequence of tooth deterioration. Bartlett et al. [8] proposed the Basic Erosive Wear Examination (BEWE) index as a simpler alternative. The recently proposed Anterior Clinical Erosive (ACE) index by Vailati and Belser [9] not only assesses the degree of wear but also provides guidance on the restoration of affected teeth.

Tooth surface loss impacts the quality of life, causing difficulties with appearance, function, sensitivity, discomfort, pulpal exposure, and potential tooth loss [10]. Nevertheless, data on treatment options for pathological TSL remain limited, posing uncertainties on treatment outcomes [11].

Preventing tooth surface loss is paramount, with early detection playing a pivotal role. As reported by Poyser et al. [12], treatment options for tooth wear depend on the position and amount of residual tooth tissue available. Traditional restorations, such as crowns, are likely to result in a greater degree of tissue loss and a significant biological impact on the teeth being restored [13]. However, innovations in adhesive dentistry have made a variety of less invasive alternatives viable [14].

Biomimetic dental materials, including composite resin restorations and partial coverage ceramics, mimic the quality of natural teeth and require minimal tooth preparation, which is useful [15].

Because no retentive characteristics are required for the restorative treatment of TSL, maximum residual tooth structure is preserved [16]. Polymerization shrinkage on composite resins can impact bonding interfaces, resulting in microleakage, secondary caries, and postoperative sensitivity [17]. Various strategies have been proposed to minimize polymerization shrinkage, including incremental placement and increasing filler content [18] with mixed outcomes.

Direct composite resins (DCR) and indirect composite resin (ICR) materials and techniques are both used in the restoration of anterior teeth. [19]. Both are technique, moisture, and contaminant-sensitive. [20]. Common techniques for isolation include cotton rolls, saliva ejector suction, and rubber dams, which enhance this outcome [21].

Bevenius et al. [22] reported the application of composite resin to address cases of tooth wear, with the direct composite being widely acknowledged as an appropriate treatment option for localized anterior tooth wear [22]. Nonetheless, the multifactorial and differing assessments on survival and success hugely influence reported outcomes and, thereby, views on treatment effectiveness [23].

This scoping review evaluates current evidence on the use of direct and indirect composites for anterior tooth wear. It discusses the survival of restorations, aesthetic outcomes, and patient satisfaction. The review also compares direct and indirect anterior composites and investigates the impact of confounding variables.

The research gap identified in this review pertains to the need for meaningful investigations that address heterogeneity and inconsistency among many studies, provide high-quality evidence through well-designed RCTs with longer follow-up periods, assess the long-term efficacy and cost-effectiveness of composite restorations, and adhere to standardized reporting guidelines. 

Addressing these gaps could contribute to a better understanding of the role of composite restorations in managing anterior tooth wear and guide clinical practice.

## 2. Materials and Methods

### 2.1. Primary Aim and Null Hypothesis

We investigated the viability of direct and indirect anterior composite restorations to manage anterior tooth surface loss.

Null hypothesis: there is no significant difference in the performance and effectiveness of direct and indirect anterior composite restorations for the treatment of anterior tooth surface loss.

### 2.2. Protocol Development

A focused question was formed to evaluate the potential of composite restorations as a treatment option for tooth surface loss in anterior teeth. The search approach was based on the PICO framework (Population, Intervention, Comparison, and Outcome) in Table 1.

### 2.3. Search Strategy

Medline, Embase, Web of Science, and the Cochrane Library search engines were used. The search used a combination of MeSH terms and focused keywords. The papers were limited to those written in English and published between January 2000 and June 2023. The inclusion and exclusion criteria are summarized in Table 2.

Table 3 itemises the search engines and search terms that were used during the literature searches. This includes the use of Boolean terms AND & OR.

### 2.4. Study Selection and Data Extraction

A manual search of the reference lists of relevant publications was conducted in addition to scanning electronic databases.

**Figure 1:**
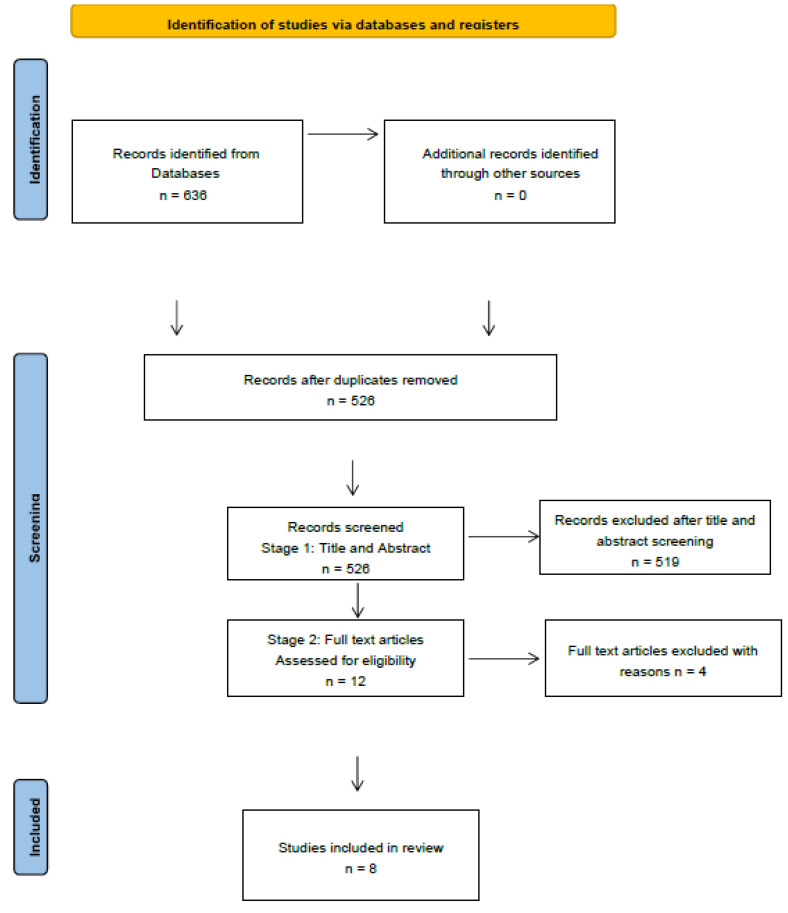
PRISMA flow diagram.

The screening of potential articles to include in the analysis was undertaken in two phases: firstly, the titles and abstracts were reviewed in line with the inclusion/exclusion criteria, and secondly, the full text of potentially relevant articles was read (Figure 1). Table 4 contains a list of the studies that were included. The extracted data from the included studies are displayed in Table 5. In this study, each arm of the randomized controlled trial (RCT) was treated as a separate study, which accounts for the presentation of 9 studies in the results, while PRISMA shows 8 studies.

Short-term survival was defined as 2 years with 95.6% survival [24].

Medium-term survival was defined as 5 years with 88.6% survival [25].

Long-term survival was defined as ≥8 years with a 92.5% survival [26].

Where some data were either missing or presented in an unusable format, the corresponding author was contacted to obtain any further information or clarification. Meta-analysis was carried out, and forest plots served as a representation of the results. The studies’ quality was examined, and any possibility of publication bias was considered as part of this scoping review.

The survival rates were used as a quantitative measure to assess the performance and longevity of dental restorations across different studies.

### 2.5. Analysis

Statistical analysis, including meta-analysis, was conducted using STATA software 18 (StrataCorp. College Station, Texas, USA 17.0/20 April 2021). To address the minor modification of zero responses, the standard procedure involves adding a small value of 0.005 to enable analysis. However, this modification can affect the results, requiring careful consideration.

Despite the software displaying negative values in the graphic, the text reports them as attenuated to zero.

Table 4 represents the authors and titles of the analyzed papers; each paper was assigned a reference number that is used throughout the subsequent text.

**Table 4:** Included studies.

Author	Title
Crins et al. (2021) [27]	Randomized controlled trial on the performance of direct and indirect composite restorations in patients with severe tooth wear
Mehta et al. (2021) [25]	Clinical performance of direct composite resin restorations in a full mouth rehabilitation for patients with severe tooth wear: 5.5-year results
A. Milosevic and G. Burnside (2016) [26]	The survival of direct composite restorations for the management of severe tooth wear including attrition and erosion: a prospective 8-year study
A. Aljawad and J. S. Rees (2016) [24]	Retrospective study on survival and patient satisfaction with composite Dahl restorations in the management of localized anterior tooth wear
Al-Khayatt et al. (2013) [28]	Direct composite restorations for the worn mandibular anterior dentition: a 7-year follow-up of a prospective randomized controlled split-mouth clinical trial
Poyser et al. (2007) [12]	The evaluation of direct composite restorations for the worn mandibular anterior dentition—clinical performance and patient satisfaction
A. M. Gow and K. W. Hemmings (2002) [29]	The treatment of localized anterior tooth wear with indirect Artglass restorations at an increased occlusal vertical dimension. Results were presented after 2 years
Hemmings et al. (2000) [30]	Tooth wear treated with direct composite restorations at an increased vertical dimension: results were provided at 30 months

### 2.6. Quality Assessment

The methodological index for non-randomized studies (MINORS) was used to assess the quality of included studies. Eight themes were graded, with a further four more in the case of comparative studies. The global ideal score was 16 for non-comparative studies and 24 for comparative studies [31].

The articles were co-rated by two authors independently (SR, PF) after a full-text reading. There were differences in ratings, and a consensus meeting was held to address and minimize these differences. As a result of the meeting, a gap or disparity in ratings between different raters was reduced from 0 to 1. 

Four of the studies were considered to be of reasonable quality (MINORS score greater than 10/16, [24,28,29,30]. Four studies were considered to be of good quality (MINORS score greater than 12/16 and 17/24) [12,25,26,27].

### 2.7. Assessment of Risk of Bias in Included Studies

**Figure 2:**
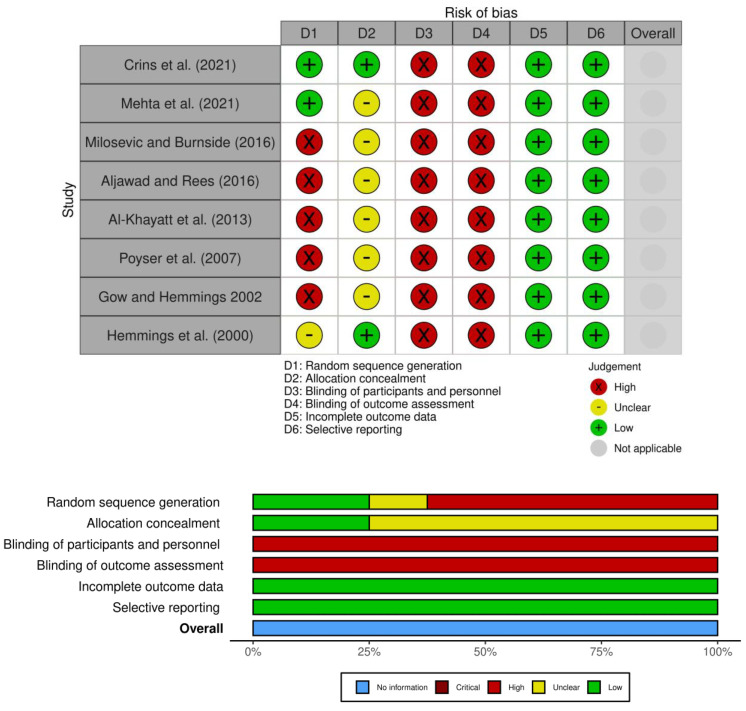
Risk of bias analysis: opinions of reviewers on each item for each included study [12,24,25,26,27,28,29,30].

A bias risk assessment was carried out by two authors (SR, PF) independently after full-text reading (see Figure 2). All the studies reported high performance and detection bias, as blinding could not be implemented for patients or operators. In general, there was an uncertain or high risk of bias for at least two of the items reported in each study.

## 3. Results

### Study Characteristics

This review analyzed eight articles that focused on direct and indirect composite restorations for the treatment of worn anterior teeth. The studies used different types of composites and evaluated various factors, such as the incisal relationship and an increase in occlusal vertical dimension. The studies were conducted in various centers, including dental hospitals and teaching centers, with varying numbers of operators. The articles analyzed mainly utilized modified versions of the United States Public Health Survey (USPHS) evaluation to assess restoration performance. Restorations were classified into three categories (A, B, and C) based on quality. Further complications were introduced when some authors used their own criteria, including severe deficiencies that required replacement, localized deficiencies that were repaired, and small material chippings that were refurbished. Most authors considered restorations requiring minor intervention as clinically acceptable for survival, while major fractures, secondary caries, or tooth loss were considered failures.

The studies assessed clinical outcomes, including discoloration, staining, and recurrent caries. The use of splints varied among the studies, and there was a noticeable variation in the reporting of restoration survival rates.

The characteristics of the included studies and confounding factors are summarized in Table 5 and Table 6.

**Table 5:** Summary of included studies.

Author Study and Follow Up Period	Direct or Indirect	Type of Composite	No. of Patients	No. of Restorations	Outcome	Conclusion
Crins et al. (2021) RCT 3 years [27]	Direct and Indirect	Micro-hybrid composite (Clearfil AP-X, Kuraray) for load-bearing areas Nano-hybrid composite (IPS Empress Direct, Ivoclar Vivadent) for buccal veneers	41	132 Direct maxilla palatal veneer 112 Indirect palatal veneer	Direct 2 repairs and 1 refurbishment on anterior palatal veneers Survival 98.4% Indirect 6 repairs on anterior palatal veneers Survival 94.6%	In patients with significant tooth wear, an indirect composite should not be used. After a 3-year monitoring period, indirect composite restorations in anterior teeth and direct composite restorations for both anterior and posterior teeth were performed satisfactorily.
Mehta et al. (2021) Prospective 5.5 years [25]	Direct	Clearfil AP-X)	34	676 anterior	77 failures (level 1 + level 2 failures) Survival 88.6%	For the treatment of severe, generalized tooth wear, a direct resin composite can provide a viable medium-term solution. The risks of failure were considerably greater for restorations requiring two sessions.
Milosevic and Burnside (2016) Prospective 8 years [26]	Direct	Hybrid composite—spectrum	164	903 661 upper 6 anterior teeth 242 lower 6 anterior teeth	67 (6.9%) of the direct composites failed. Failure was defined as a total debond or chip. Survival 92.5%	Composite is a suitable material for restoration. Clinical significance: according to this study, posterior occlusal support is critical to enhance longevity.
Aljawad and Rees (2016) Retrospective 25.4 months [24]	Direct	CeramX Duo)	41	296	Major failure 13 bulk fractures (4.4%) Minor failure 6 chippings; 14 marginal discoloration (8.7%); Success rate of 88.8%; Survival rate of 95.6%.	There was high-patient satisfaction. Clinical relevance: a good short- to medium-term survival rate is achieved with composite restorations at an increased OVD to manage localized anterior tooth wear.
Al-Khayatt et al. (2013) Prospective 7 years [28]	Direct	Micro-hybrid–Herculite XRV composite and Optibond dentine bonding agent	15	85	Survival of 85/89 at the 7-year (85%)	For the majority of patients, direct composite restorations worked well, provided high levels of patient satisfaction, and required minimal maintenance.
Poyser et al. (2007) Prospective 2.5 years [12]	Direct	Micro-hybrid–Herculite XRV composite and Optibond dentine bonding agent	14	73	Survival of 72/77 at 2.5 years. (94%) 6% complete failure; 19% mild staining; 26% marginal discoloration; 54% defective marginal adaptation.	A simple and quick way to manage the worn mandibular teeth is to place direct composite restorations at an increased occlusal vertical dimension. A high degree of patient satisfaction is maintained throughout the medium term, with good patient acceptance and adaption to the approach.
Gow and Hemmings 2002 Prospective 2 years [29]	Indirect	Artglass restoration	12	75	100% survival 10 restorations had minor failures requiring chairside repairs.	Artglass indirect palatal restorations are an effective short-term treatment for localized anterior teeth wear.
Hemmings et al. (2000) Prospective 30 months [30]	Direct	In group A, Durafill composite and Scotch bond Multipurpose dentine adhesive (N = 52). In group B, Herculite XRV composite and Optibond dentine bonding agent (N = 52)	16	104	93% Survival Group A Repair—24 Replace–9 Group B Repair—4 Replace–2	For the treatment of localized anterior tooth wear, direct composite restorations could be an option.

**Figure 3:**
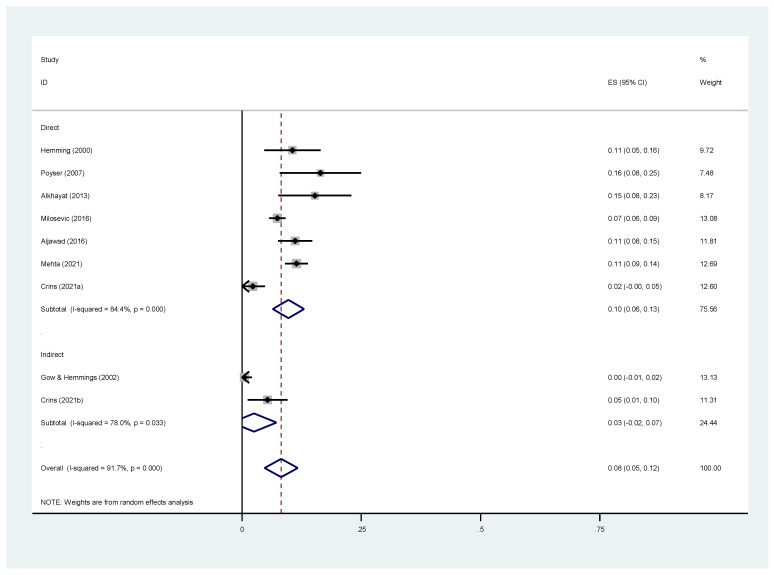
Forest plot for the survival rate of direct and indirect composite restorations [12,24,25,26,27,28,29,30].

Figure 3 is a forest plot that categorizes studies into “direct” and “indirect” groups. Both groups show small effect sizes, with pooled estimates of 0.10 (95% CI 0.06 to 0.13) for “direct” and 0.03 (95% CI 0.02 to 0.07) for “indirect”. There was significant heterogeneity within both groups, with I ^2^ values of 84.4% and 78% for “direct” and “indirect,” respectively.

The overall pooled estimate for both groups combined was 0.08 (95% CI 0.05 to 0.12), suggesting an 8% failure rate, but the wide confidence interval (5% to 12%) made it unreliable because of heterogeneity. The overall heterogeneity was high at 91.7% (*p* < 0.001), indicating a substantial variation in the study results, making firm conclusions challenging.

Comparing the “direct” and “indirect” groups, the difference was not statistically significant. High heterogeneity (91.7%) adds uncertainty to any potential differences between the two groups, drawing implications for this scoping review.

To identify sources of heterogeneity, subgroup comparisons for potential confounders were conducted. This analysis acknowledges limitations due to considerable heterogeneity, indicating the presence of unaccounted-for factors influencing the results.

A clear pattern emerged in the subgroup analysis as follows: the use of rubber dams and splints seemed to increase the heterogeneity in the results. This suggests that these confounding factors might introduce more diversity in these study outcomes. Investigating the reasons behind this is crucial as it provides insights into how rubber dams and occlusal splints affect the results differently (Table 7).

## 4. Discussion

This scoping review highlights the need for more compelling research to support the application of specific materials or procedures in the restoration of worn teeth despite its frequent occurrence in clinical practice. The articles included in this scoping review that assessed the longevity of composite restorations had limitations in terms of sample size and follow-up duration. The use of multiple operators and the comparison of various materials further contributed to the ambiguity of these results.

The clinical studies analyzed in this review encompassed a wide range of clinical presentations involving worn dentition, including attrition, erosion, and abrasion, which required different management approaches. This variation in patient characteristics adds uncertainty to the assessment of results. Moreover, differences in defining and interpreting failure and adverse events across these studies made direct data comparison challenging. The lack of consistency among the studies contributed to reported degrees of heterogeneity, making it virtually impossible to provide a definitive recommendation for one material or technique over another.

Another significant challenge encountered in data extraction was the aggregated reporting of restoration failures. Determining the precise number of direct or indirect anterior composites that were successful proved difficult. For example, Gulamali et al. [32] reported 283 composite resin restorations in 26 patients, but they did not differentiate between the number of direct and indirect composites that lasted during the 10-year evaluation. Consequently, no meaningful inferences could be drawn, and attempts to contact the authors for clarification were unsuccessful, resulting in the exclusion of this study from our analysis. Similar variations in data reporting were also discussed in other systematic reviews [33,34], raising questions about the reliability of the research.

The analysis suggests a marginally lower failure rate in indirect restorations. However, this finding may not be a true reflection of the actual situation due to the limited number of studies (only 2) that examined indirect restorations. The small sample size may not provide a comprehensive and reliable representation of the true effect. The forest plot analysis suggests small effect sizes in both “direct” and “indirect” groups, but the overall estimate’s uncertainty and substantial heterogeneity (I^2^ = 91.7%) require further investigation to draw definitive conclusions.

Confounding factors that contributed to heterogeneity were observed in the findings of this scoping review. Factors such as the type of tooth wear, the use of a rubber dam, splints, length of the follow-up period, incisal relationship, outcome measures, and operators potentially influenced the results. The etiology of tooth wear in the patients included in the trials was clearly determined and could confound the research findings. In addition, the intensity and forms of tooth wear, as well as the clinical condition of teeth undergoing restoration, were often inadequately reported in the selected studies. Different types of tooth wear, such as erosion and bruxism, can have varying effects on restorations [35]. Patients with severe bruxism may exhibit parafunctional behaviors that can pose clinical challenges for any restorative material when bruxism is a primary etiological factor. The forces that initially caused the teeth to wear will also affect the composite restorations, as shown in Milosevic and Burnside’s [26] research, where the attrition group had a higher percentage of failures compared to the erosion group. The reporting of the cause of wear varied across studies, adding to the heterogeneity observed in this scoping review.

The methodological variations identified below also had a huge impact on the heterogeneity noted. 

The type of composite used: filler content and load. Among the included studies, different composites were used, ranging from micro-filled, micro-hybrid, and nanohybrid. Although highly polishable, micro-filled composites have higher thermal expansion, greater water sorption, and limited hardness compared to hybrid composites because the amount of resin in it is larger and the filler concentration is lower [36]. 

However, it is worth noting that the lack of standardization and significant variation among studies makes it challenging to compare results based on the type of composite used.

Occlusal splints: literature indicates that splints can be used both to prevent tooth wear and preserve reconstructed teeth. Bartlett and Sundaram [37] demonstrated that the absence of an acrylic splint may contribute to a higher incidence of fractures. In the systematic review by Crins et al. [27], patients who received an occlusal splint following treatment had a survival rate of 97.2%. However, Aljawad and Rees [24] reported a survival rate of 95.6% without mentioning the use of occlusal splints. The subgroup analysis suggested the absence of rubber dams and splints that could decrease heterogeneity in these studies, but it could also indicate methodological weaknesses. This requires further research.

Type of isolation: achieving the optimal bonding of composite restorations requires complete isolation and a contaminant-free environment. The use of a rubber dam has been shown to provide excellent isolation and improved visibility, significantly reducing challenges during composite restoration placement. However, the results of Milosevic and Burnside’s [26] study, which did not utilize a rubber dam, reported high survival rates. By contrast, Bartlett and Sundaram’s [37] study, which used a rubber dam, reported very high failure rates. Loomans et al. [38] reported good survival rates without explicitly mentioning isolation techniques. Drawing conclusive findings is challenging due to the failure of researchers to describe their isolation techniques. Additionally, other confounding variables, such as the absence of post-treatment splints, could contribute to higher failure rates despite the use of a rubber dam. 

Layering technique: the layering technique refers to how the composite material is applied in multiple layers during the restoration procedure. The choice of layering technique can affect the appearance, strength, and overall success of the restoration. Studies have not mentioned the manner in which the composite was layered and cured. Differences in layering techniques could contribute to inconsistencies in the study findings.

The length of the follow-up period varied from 2 to 8 years. It is common sense to expect that trials with a longer follow-up period demonstrate a higher degree of intervention. However, the Forest plot did not reflect this outcome. The analysis of studies with a follow-up of three years or less compared to studies with a follow-up of three years or more revealed that significant failures became less frequent as time passed. Studies with greater failure rates often reported any need for intervention as ‘bad’, while studies with lower intervention rates reported only a subset of events. However, due to significant heterogeneity, a clear comparison and conclusion cannot be drawn regarding this aspect of the scoping review.

To ensure consistency and comparability across studies, it is crucial to provide detailed information about the methodology. Addressing and standardizing these methodological factors in future research can contribute to a more robust and reliable body of evidence regarding the use of composite restorations for anterior tooth surface loss.

When repairing worn-out dentition, a stronger composite is critical. Hybrid composites have the advantage of using a range of filler particles consisting of submicron particles 0.4 μm to large particles of 1.0 μm in size. While bigger fillers offer higher strength and lessen expansion/contraction, smaller particles offer wear resistance and lessen shrinkage stress. Therefore, a hybrid composite is advised for the treatment of tooth wear [36]. To ensure optimal bonding and procedural effectiveness, the isolation of teeth and moisture management are essential. Techniques such as suction, rubber dams (dry), and cotton wool rolls can be employed to achieve this [39]. While the literature suggests that air abrasion can enhance bond strength without compromising tooth structure, none of the included studies utilized this method [40]. Three-step etch and rinse adhesives performed better than self-etch resins, which were susceptible to water deterioration [41]. Studies also suggest that heating the composite resin can enhance its physical properties, including flexural strength, wear resistance, and elastic modulus, as well as reduce the occurrence of cavities due to improved flow characteristics [42]. In conclusion, a recommended technique for treating tooth wear involves sandblasting the teeth to promote bonding, isolating the area with a rubber dam, separately etching and cleaning the enamel and dentin, and applying a heated hybrid direct composite.

Although there is potential promise in using glass fiber-reinforced composite resins with improved wear resistance and minimal shrinkage for treating tooth wear, there is currently no published evidence confirming their efficacy [43].

## 5. Conclusions

The studies included in this scoping review collectively suggest that composite restorations can be considered a viable short to medium-term treatment option for addressing anterior tooth surface loss. While some studies reported favorable outcomes for composite restorations, it is important to acknowledge the limitations and inconsistencies within the reviewed literature. 

The evidence, though not statistically significant, marginally supports the use of anterior composite restorations for managing tooth wear in the short-to-medium term. However, to definitively establish their long-term efficacy, further research is imperative, particularly in the form of well-designed randomized controlled trials with standardized protocols and extended follow-up periods. These efforts could enable more reliable clinical conclusions and guide evidence-based decision-making in the treatment of anterior tooth wear.

## Figures and Tables

**Table 1:** PICO framework.

Population	Patients with Anterior Tooth Surface Loss
Intervention	Restoration of worn teeth with direct anterior composite resin
Comparison	Restoration of worn teeth with indirect anterior composite resin
Outcome	Survival of direct and indirect anterior composite used in tooth surface loss

**Table 2:** Inclusion and exclusion criteria.

Inclusion Criteria	Exclusion Criteria
Publication date: 2000–2023 Restoration follow-up period for a minimum of one year English language Randomized and non-randomized controlled trials, retrospective, and prospective studies Direct and indirect anterior composite restoration	Animal studies Studies on deciduous teeth Adults with Class V abrasion cavities Studies involving cast restorations—PFM crown, all ceramic restorations, cast gold restorations Case studies and case series Posterior composites

**Table 3:** Electronic databases and search terms.

Electronic databases and search terms	Ovid MEDLINE, Embase Classic + EMBASE, The Cochrane Library, Web of Science tooth wear OR dental wear OR tooth attrition OR dental abrasion OR dental erosion AND composite resin OR composite AND Dental restoration failure OR Dental Stress Analysis OR survival OR Longevity OR Fracture OR Chipping (Tooth or teeth or dental) near/3 (wear* or attrition or erosion or abrasion) (Topic) and “composite resin*” or “direct composite*” or “indirect composite*” or “dental restoration*” (Topic) and survival or “success rate*” or longevity or fracture* or chip* or “survival analysis” or “dental restoration failure” or “dental stress analysis” (Topic)
Other strategies used to find specific research	Additional articles were found by hand searching reference lists of pertinent papers and reviews.
Journals hand-searched	British Dental Journal, Journal of Aesthetic Dentistry, Journal of Adhesive Dentistry, Journal of prosthetic dentistry, Journal of Oral Rehabilitation would be searched manually

An asterisk (*) serves as a truncation symbol, allowing to encompass all possible word endings in a search.

**Table 6:** List of confounding factors.

Author	Isolation	Splint	Assessment Criteria
Crins et al. (2021) [27]	Rubber dam or cotton roll	Not soon after the treatment 3 patients had a 1-year recall, as several fractures were seen	“(F1) severe deficiencies that were replaced or in case of loss of the tooth; (F2) Restorations with localized deficiencies repaired; (F3) Restorations with small material chippings that needed refurbishment by polishing”
Mehta et al. (2021) [25]	Rubber dam	Acrylic splints were not initially recommended, but when failure was later observed owing to fracture or wear from bruxist tendencies, a night guard was then indicated.	“Level 1 failure’ = a restoration with a severe deficiency, that required restoration replacement (to include the need for endodontic treatment, or a dental extraction); ‘Level 2 failure’ = a restoration with localized deficiencies, that was repaired, and ‘Level 3 failure’ = a restoration with small material chips”
Milosevic and Burnside (2016) [26]	No rubber dam	Not mentioned	Defined “failure as total debond or chip”
Aljawad and Rees (2016) [24]	Rubber dam not used	Not mentioned	Modified USPHS
Al-Khayatt et al. (2013) [28]	Rubber dam used	Not given	Modified USPHS
Poyser et al. (2007) [12]	Rubber dam used	Not given	Modified USPHS
Gow and Hemmings (2002) [29]	Not mentioned	Not mentioned	Modified USPHS (United States Public Health Services)
Hemmings et al. (2000) [30]	Rubber dam	Not given	Fracture, marginal discoloration, loss of marginal integrity, obvious wear, discomfort or sensitivity, endodontic failure, and cosmetic failure were all indications of failure.

**Table 7:** Sub-group analyses to investigate heterogeneity.

	I Squared % (N Studies)	*p*-Value
**Method**		
Direct [12,24,25,26,27,28,30]	84.4% (7)	<0.001
Indirect [27,29]	78% (2)	<0.001
Both [27]	N/A (1)	N/A
**DIRECT ONLY**		
**Design**		
Prospective [12,25,26,27,28,30]	85.9(6)	0.012
Retrospective [24]	N/A (1)	N/A
**Follow-up**		
Less than 3 years [12,24,27,30]	87.5 (4)	<0.001
More than 3 years [25,26,28]	79.4 (3)	<0.001
**Occlusal Splint**		
Used [25,27]	96.5 (2)	<0.001
Not used [12,24,26,28,30]	61.4 (5)	0.035
**Rubber Dam**		
Used [12,25,27,28,30]	88.6 (5)	<0.001
Not used [24,26]	70.5 (2)	0.066

## Data Availability

All data used in this study are available on the websites searched including MEDLINE, Embase, Web of Science and the Cochrane library.
